# The degree of cardiac baroreflex involvement during active standing is associated with the quality of life in fibromyalgia patients

**DOI:** 10.1371/journal.pone.0179500

**Published:** 2017-06-14

**Authors:** Antonio Roberto Zamunér, Alberto Porta, Carolina Pieroni Andrade, Meire Forti, Andrea Marchi, Raffaello Furlan, Franca Barbic, Aparecida Maria Catai, Ester Silva

**Affiliations:** 1 Department of Physical Therapy, Federal University of Sao Carlos, Sao Carlos, Brazil; 2 Department of Physical Therapy, Universidade do Sagrado Coração, Bauru, Brazil; 3 Department of Biomedical Sciences for Health, University of Milan, Milan, Italy; 4 Department of Cardiothoracic, Vascular Anesthesia and Intensive Care, IRCCS Policlinico San Donato, San Donato Milanese, Milan, Italy; 5 Department of Electronics Information and Bioengineering, Politecnico di Milano, Milan, Italy; 6 Internal Medicine, Humanitas Research Hospital, Humanitas University, Rozzano, Italy; Ospedale del Cuore G Pasquinucci Fondazione Toscana Gabriele Monasterio di Massa, ITALY

## Abstract

Fibromyalgia syndrome (FMS) is a rheumatologic disorder characterized by chronic widespread pain, fatigue and other symptoms. Baroreflex dysfunction has been observed in women with FMS. However, it is unknown whether the limited involvement of the baroreflex control during an orthostatic stimulus has some impact on the quality of life of the FMS patient. Therefore, the aim of the study is evaluate the relationship between the quality of life of the FMS patient and indexes of the cardiovascular autonomic control as estimated from spontaneous fluctuations of heart period (HP) and systolic arterial pressure (SAP). We enrolled 35 women with FMS (age: 48.8±8.9 years; body mass index: 29.3±4.3 Kg/m^2^). The electrocardiogram, non-invasive finger blood pressure and respiratory activity were continuously recorded during 15 minutes at rest in supine position (REST) and in orthostatic position during active standing (STAND). Traditional cardiovascular autonomic control markers were assessed along with a Granger causality index assessing the strength of the causal relation from SAP to HP (CR_SAP→HP_) and measuring the degree of involvement of the cardiac baroreflex. The impact of FMS on quality of life was quantified by the fibromyalgia impact questionnaire (FIQ) and visual analog score for pain (VAS pain). No significant linear association was found between FIQ scores and the traditional cardiovascular indexes both at REST and during STAND (p>0.05). However, a negative relationship between CR_SAP→HP_ during STAND and FIQ score was found (r = -0.56, p<0.01). Similar results were found with VAS pain. In conclusion, the lower the degree of cardiac baroreflex involvement during STAND in women with FMS, the higher the impact of FMS on the quality of life, thus suggesting that Granger causality analysis might be clinically helpful in assessing the state of the FMS patient.

## Introduction

Fibromyalgia syndrome (FMS) is a rheumatologic disorder characterized by chronic widespread pain, fatigue and other symptoms that might be related to dysautonomia (i.e., insomnia, irritable bowel, anxiety, depression) [1]. As a consequence, autonomic function and cardiovascular control have been frequently assessed in FMS and their evaluation has been typically based on the analysis of spontaneous fluctuations of heart period (HP) and systolic arterial pressure (SAP) [2–6]. These studies have reported that patients with FMS feature an increased sympathetic control and a reduced vagal modulation in supine position at rest (REST) [4,7,8], a lower cardiac baroreflex sensitivity compared to age-matched healthy subjects at REST [4,7,8], an inability of reducing further baroreflex sensitivity during an orthostatic challenge such as active standing (STAND) [6] and a limited involvement of the baroreflex control in regulating arterial blood pressure during STAND as detected by Granger causality analysis [6]. However, whether all these parameters derived from HP and SAP variabilities are related to the quality of life of the FMS patient, as assessed from a clinical score such as the fibromyalgia impact questionnaire (FIQ) and the visual analog score for pain (VAS pain), is unknown.

We hypothesize that FMS patients with higher FIQ and VAS pain scores, indicating a poor quality of life, are those with lower degree of cardiac baroreflex involvement during STAND as a manifestation of their inability to deal with this common task in their daily life.

Therefore, the present study aims to evaluate the relationship between the quality of life of FMS women and indexes of the cardiovascular autonomic control estimated from spontaneous HP and SAP variabilities. This analysis is not only limited to traditional markers derived from HP and SAP variabilities but it includes even those quantifying the strength of the causal relation along a given time direction (e.g. from SAP to HP along cardiac baroreflex) computed according to Granger causality analysis.

## Materials and methods

The study was approved by the Ethics in Research Committee from Federal University of Sao Carlos (protocol number 112.508). All participants gave written informed consent.

### Enrolled population

From January 2013 to December 2015, 35 women with clinical diagnosis of FMS took part in the study (age: 48.8±8.9 years; body mass index: 29.3±4.3 Kg·m^-2^). The diagnosis was made by a board certified rheumatologist according to criteria established by the American College of Rheumatology [9]. The subjects were recruited from local community among those who responded to flyers posted in university buildings, orthopedic and rheumatologic clinics, or belonged to our database of FMS patients. The enrolled subjects had no history of cardiovascular, respiratory or metabolic disease, inflammation, neurological disorders and cognitive deficits preventing the regular progress of the study. They were not smokers, or engaged in regular physical activity, or make continuous use of drugs or alcohol.

### Experimental procedure

All experiments were carried out in the morning (8 a.m. to 12 p.m.) to minimize the impact of the circadian rhythm on cardiovascular variables. Room temperature was maintained at 22°C and the relative air humidity between 40% and 60%. Participants were acquainted with the experimental protocol and were instructed to abstain from stimulants (coffee, tea, soft drinks) and alcoholic beverages for the 24 hours preceding the examination, and to have a light meal at least two hours before the test. Subjects were asked to avoid strenuous physical activity in the two days before the tests. Participants took no psychotropic drugs or other medications known to alter autonomic activity for at least 4 weeks before the study, including beta-blockers, antihypertensive drugs, tranquilizers, or antidepressants. The participants with regular menstrual cycle (28 ± 2 days) were assessed during the follicular phase, i.e., 7–10 days after the start of menses.

We recorded surface ECG (modified lead I) (BioAmp FE132, ADInstruments, Australia), noninvasive blood pressure (Finometer Pro, Finapres Medical Systems Ohmeda, Amsterdam, Netherland), and respiratory activity via a piezoelectric respiratory belt (Thoracic Belt, Marazza, Monza, Italy) from every subject. The arterial pressure signal was cross-calibrated in each session by measuring the blood pressure with a sphygmomanometer at the onset of the session. Signals were sampled at 1000 Hz using a commercial device (BioAmp Power Lab, AD Instruments, Australia).

Data acquisition was performed for 15 minutes at REST and during STAND. Before beginning data acquisition, about 20 minutes were allowed for hemodynamic stabilization.

### Extraction of the beat-to-beat variability series

Standard procedures were followed to build the beat-to-beat HP and SAP variability series [6]. Briefly, HP was measured from the ECG as the temporal distance between two consecutive R-wave peaks. The maximum arterial pressure value was identified in the HP and taken as SAP. Respiratory (RESP) series was obtained by sampling the respiratory signal at the R-wave peak. Series lengths of 256 consecutive measures were extracted from the entire HP, SAP and RESP sequences both at REST and during STAND. Stationarity of the HP and SAP series was checked according to the test proposed in [10].

### Power spectral density estimation

Power spectrum was estimated according to a parametric approach fitting the series with an autoregressive (AR) model [11]. AR spectral technique provided a factorization of the spectral density into components that were classified according to their central frequency. If the central frequency belonged to the low frequency (LF, from 0.04 to 0.15 Hz) band or to the high frequency (HF, from 0.15 to 0.5 Hz) band, the corresponding component was labeled as LF or HF respectively. The LF, or HF, power was computed as the sum of the powers of all LF, or HF, components.

### Frequency domain assessment of cardiac baroreflex sensitivity

The cardiac baroreflex sensitivity was computed as the square root of the ratio of the power of HP to that of SAP. The ratio was calculated in both LF and HF bands [11] and indicated as α_HF_ and α_LF_ respectively. Two parameters were considered as prerequisites for the reliable estimation of cardiac baroreflex sensitivity [11]: i) the squared coherence function K^2^_HP-SAP_ should be higher than 0.5 at the considered central frequency as an indication of a significant HP-SAP correlation; ii) the phase of the cross-spectrum Ph_HP-SAP_ should be lower than 0 at the considered central frequency as an indication that HP changes lag behind SAP variations [11].

### Granger causality evaluation

A Granger causality approach [12–14] was utilized to assess the strength of the directed dependence of HP on SAP via the computation of the causality ratio (CR) (CR_SAP→HP_). This index was computed over the set of variability series Ω = {HP,SAP,RESP}. SAP is said to Granger-cause HP if the HP dynamics can be better predicted in Ω than in Ω after exclusion of the presumed cause SAP (i.e., Ω\SAP = {HP,SAP}) [15]. The inclusion of RESP in Ω assures that the RESP influences are disambiguated while assessing the directionality of the HP-SAP variability interactions [16,17]. Granger approach to the evaluation of causality from SAP to HP was described in detail elsewhere [12,13]. Briefly, after defining the prediction error as the difference between the current HP value and its prediction based on a linear regression model, CR_SAP→HP_ is computed as the fractional predictability improvement of HP due to the introduction of SAP in Ω\SAP. Thus, the higher and positive the CR_SAP→HP_ is, the stronger the strength of the causal link from SAP to HP. The significance of CR_SAP→HP_ was checked by comparing the mean square prediction error of HP in Ω and in Ω\SAP via the F-test [18]. If the CR_SAP→HP_ adjusted for the degrees of freedom [17,18] was larger than the critical value of the F distribution for a significance level of 0.01, the null hypothesis of absence of a causal relation from SAP to HP was rejected and we accepted the alternative hypothesis that SAP Granger-caused HP, indicated as SAP→HP in the following. SAP→HP is taken as an indication that cardiac baroreflex is working and CR_SAP→HP_ measured its degree of involvement.

### Quality of life and pain assessment

The quality of life was assessed by the FIQ. This questionnaire was specifically developed to assess disease severity and impact of FMS on the subject’s quality of life. This questionnaire comprises issues related to functional ability, professional situation, psychological disorders and physical symptoms. The score of this questionnaire ranges from 0 to 100, and the higher the score, the greater the impact of FMS on the subject’s quality of life [19]. It was shown that the FIQ score was able to capture the overall effect of FMS and measure the individual response to the application of a clinical protocol [20,21].

A 100-mm visual analogue scale (VAS) was applied to evaluate the current intensity of overall body pain felt at rest (0 = no pain, 100 = worst pain ever felt).

### Statistical analysis

Gaussianity of the data was verified by Shapiro-Wilk test. Linear regression analysis was used to assess the association between the impact of FMS on quality of life, assessed by the FIQ, and the indexes of the cardiovascular autonomic control. Pearson’s correlation coefficient r was computed and type I error probability p was estimated. Partial correlation analyses were carried out between FIQ or VAS pain and the indexes of the cardiovascular autonomic controlling for body mass index (BMI) and disease duration. Statistical significance was set at 5% for all tests. SPSS 20.0 (SPSS, Inc, Chicago, IL) was used for all analysis.

## Results

Table 1 displays the demographic characteristics of our FMS group in resting condition. As expected, FMS patients featured pain as evident from the elevated VAS pain score and the high number of tender points. In our group BMI was larger than 25 kg/cm^2^ in 91.4% of the subjects.

**Table 1:** Demographic characteristics of the FMS population in resting condition.

Variables	FMS (n = 35)
Age (years)	48.83 ± 8.89
Weight (kg)	73.49 ± 12.34
Height (cm)	158.09 ± 5.78
BMI (kg/m^2^)	29.27 ± 4.31
HR (bpm)	72.09 ± 8.87
SAP (mmHg)	120.71 ± 12.14
DAP (mmHg)	74.29 ± 8.06
Respiratory rate (rpm)	15.86 ± 2.50
VAS pain (0–100 mm)	49.2 ± 20.6
Number of tender points (0–18)	16.97 ± 1.89
Disease duration (years)	9.20 ± 6.29

BMI: body mass index; HR: heart rate; SAP: systolic arterial pressure; DAP: diastolic arterial pressure; VAS pain: visual analog scale for pain.

**Fig 1:**
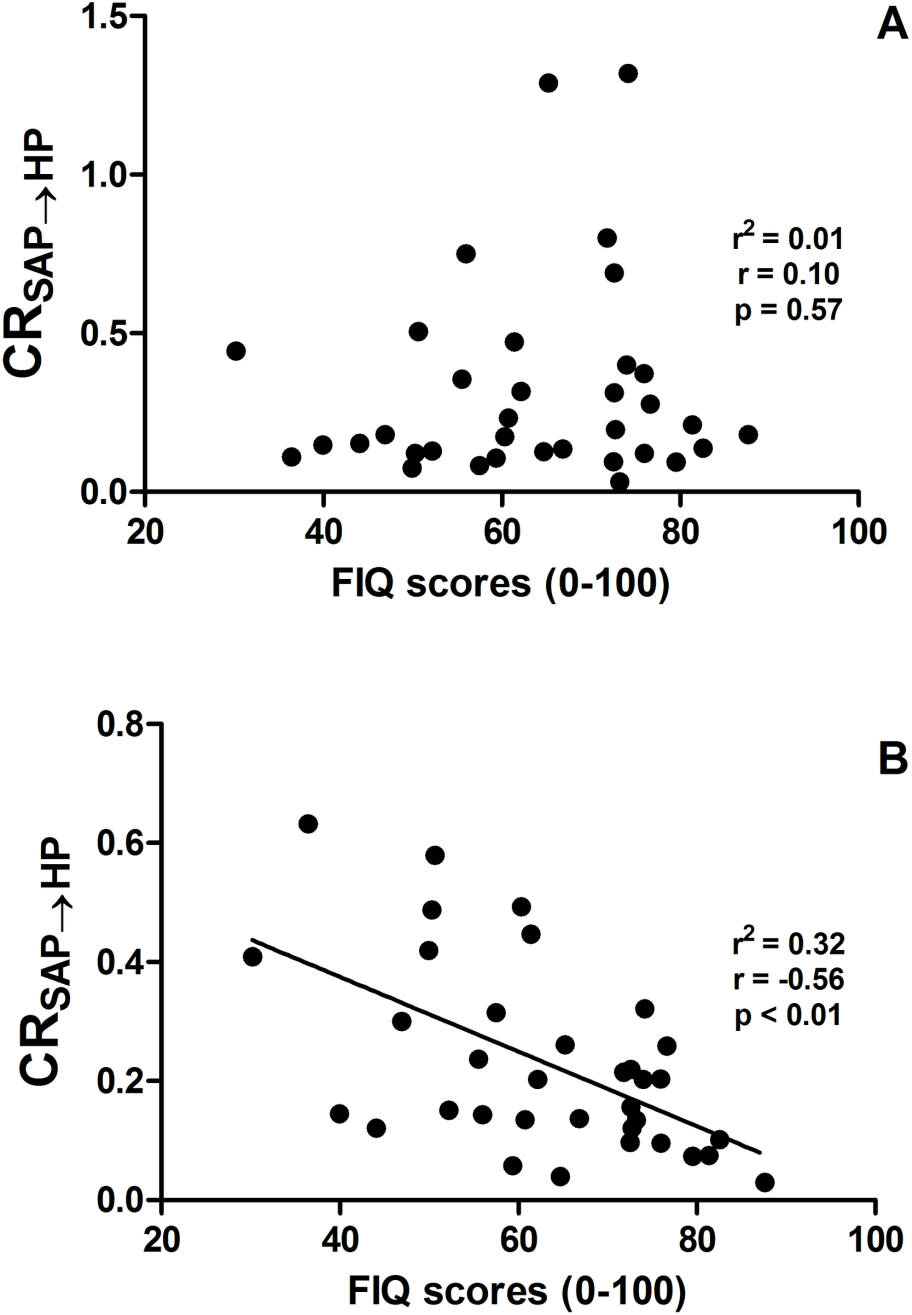
**The scatter plots of CR**_**SAP→HP**_ **on FIQ score are shown at REST (a) and during STAND (b).** Each solid circle is relevant to a pair (FIQ score, CR_SAP→HP_) computed over a single individual. The linear regression is shown as well when a significant association between the two variables is found with p<0.05.

Results of the correlation analysis between the cardiovascular control markers derived from HP and SAP variability series and the FIQ score are shown in Table 2. Traditional autonomic indexes in time and frequency domains were not significantly associated with the FIQ score both at REST and during STAND (p>0.05). CR_SAP→HP_ was unrelated to FIQ score at REST (Fig 1A). However, a significant and negative relation of CR_SAP→HP_ on FIQ score was found during STAND (r = -0.56, p<0.01, Fig 1B). A similar result was found between a marker of pain intensity in resting condition (i.e. VAS pain) and CR_SAP→HP_ (r = -0.38 with p<0.05 only during STAND). Results were confirmed even when correlation analyses were carried out by accounting for BMI (CR_SAP→HP_ and FIQ: r = -0.55, p<0.001; CR_SAP→HP_ and VAS pain: r = -0.38, p<0.03) and disease duration (CR_SAP→HP_ and FIQ: r = -0.58, p<0.0001; CR_SAP→HP_ and VAS pain: r = -0.39, p<0.02).

**Table 2:** Linear correlation analysis of the cardiovascular control parameters on the FIQ score at REST and during STAND.

Cardiovascular parameters	REST		STAND	
	r	p	r	p
μ_HP_	-0.08	0.64	0.05	0.77
σ^2^_HP_	-0.06	0.71	0.24	0.17
HF_HP_	0.07	0.69	0.34	0.04
μ_SAP_	-0.06	0.71	0.13	0.44
σ^2^_SAP_	-0.004	0.98	0.17	0.32
LF_SAP_	-0.09	0.61	0.06	0.73
α_LF_	-0.10	0.58	-0.07	0.69
α_HF_	-0.05	0.77	0.19	0.26
CR_SAP→HP_	0.10	0.57	-0.56	<0.01

HP: heart period; SAP: systolic arterial pressure; LF: low frequency; HF: high frequency; μ_HP_: HP mean; σ^2^_HP_: HP variance; HF_HP:_ HF power of HP series expressed in absolute units; μ_SAP_: SAP mean; σ^2^_SAP_: SAP variance; LF_SAP_: LF power of SAP series expressed in absolute units; α_LF_: baroreflex sensitivity assessed in the LF band; α_HF_: baroreflex sensitivity assessed in the HF band; CR_SAP→HP_: Granger causality ratio from SAP to HP; r: Pearson’s correlation coefficient; p: probability of type I error.

## Discussion

The main findings of the present study are that the FMS patients with lower degree of cardiac baroreflex involvement during STAND (i.e. with Granger causality ratio from SAP to HP close to zero) were those with higher impact of FMS on quality of life and with higher pain in resting condition. These findings corroborate our hypothesis that lower degree of baroreflex involvement during an orthostatic stressor could be negatively related to the quality of life. Remarkably, more traditional univariate and bivariate cardiovascular control indexes derived from HP and SAP variability series were not significantly related to the quality of life of the FMS patient.

To our knowledge, this is the first study assessing the association between a Granger causality index and a clinical measure of the quality of life, as assessed by the FIQ score, in the FMS patient. The CR_SAP→HP_ measures the strength of the causal link from SAP to HP, thus quantifying the degree of involvement of the cardiac baroreflex [14]. It is known that an orthostatic stimulus increases the involvement of cardiac baroreflex in governing HP changes [22,23]. A possible explanation relies on the unloading of cardiopulmonary and arterial baroreceptors consequent to the decrease of the central blood volume, leading to the activation of the cardiac baroreflex in the attempt to prevent the arterial pressure drop. A recent study revealed that a considerable amount of subjects with FMS featured an impaired baroreflex control during STAND, as indicated by a reduced gain of the HP-SAP relation usually referred to as baroreflex sensitivity [6]. However, while modifications of the cardiac baroreflex sensitivity in FMS are well-known as well as changes of the cardiac baroreflex solicitation, as measured by the strength of the causal relation from SAP to HP [6], it is unknown their relation with the subject’s quality of life.

Complaining about symptoms such as palpitations, fatigue and inability to cope with STAND for prolonged times is common in patients with FMS [24]. Indeed, Furlan et al [4] reported a reduced orthostatic tolerance in patients with FMS as stressed by the higher rates of syncope in patients with FMS compared to a healthy control group. Moreover, Bou-Holaigah et al. [25] reported an abnormal decrease of blood pressure during head-up tilt test in patients with FMS leading to syncope or presyncope defined as a drop in SAP of at least 25 mmHg with no associated increase in heart rate.

It is well known that orthostatic intolerance interferes with quality of life [26]. Although the FIQ does not comprise any direct question related to orthostatic intolerance or presyncope symptoms, it is likely that the difficulty in coping with this important stressor might significantly impact the perception that a subject has about his/her health state and, consequently, his/her quality of life. This perception might have an indirect impact on several answers of the FIQ questionnaire leading to higher scores. The FIQ score is considered a clinical measure of the quality of life in the FMS patient since it captures the overall effect of FMS and also the individual response to a clinical protocol devised to improve the health state of the FMS patient [20,21]. It is composed by 19 questions organized in 10 items measuring physical functioning, daily living activities, work status, depression, anxiety, goodness of sleep, pain, stiffness, fatigue, and well-being. The higher the score is, the higher the impact of FMS on quality of life and the worse the condition of the FMS patient. Therefore, a reduced orthostatic tolerance due to the cardiac baroreflex dysfunction could interfere with the FMS patient’s ability to deal with his/her daily life activities, thus supporting the possible association of cardiac baroreflex parameters with the quality of life in FMS patients. As a matter of fact, we found that the FIQ score was associated with the degree of baroreflex involvement in regulating HP-SAP dynamical interactions as assessed from the strength of causal relation from SAP to HP, even after controlling for BMI and disease duration. The lower (and the closer to zero) the strength of the causal link from SAP to HP, the higher the FIQ score and, thus, the worse the impact of FMS on the quality of life.

Another noteworthy result of this study is the negative association between the strength of causal relation from SAP to HP during STAND and a measure of the pain intensity in resting condition (i.e. VAS pain), which also remained after controlling for BMI and disease duration. Thus, the FMS women with lower strength of the causal link from SAP to HP were those with higher levels of pain in resting condition. A previous study has reported that baroreflex sensitivity and sympathetic activity were significantly associated with pain intensity in women with FMS [27]. Several mechanisms have been proposed to explain this association, such as the interactions between the autonomic and sensory systems occurring in the nucleus tractus solitarius integrating information from baroreceptor inputs and neurons implicated in pain modulation [8,27–30]. Given that pain is directly captured by a component of the FIQ and the possible association between pain and baroreflex impairment, the observed link between the quality of life and the degree of baroreflex involvement might be the reflex of the association of the quality of life with pain. However, the different degrees of association suggest that the relation between the quality of life and the baroreflex involvement might be not the mere consequence of the increased cardiovascular deconditioning with the pain intensity.

Traditional cardiovascular control indexes were found to be unrelated to the FIQ score. This finding stresses the clinical relevance to account for causality in studies assessing the cardiovascular autonomic control. The relevance of accounting for causality in FMS patients was already proved by Zamunér et al. [6] who showed a diminished cardiac baroreflex gain at REST using a model-based causal approach and a reduced involvement of cardiac baroreflex control in regulating arterial blood pressure during STAND via a Granger causality assessment. The present study confirms causality analysis conveys complementary information to traditional cardiovascular control indexes given that the unique index exhibiting a significant relation with FIQ score or pain intensity was CR_SAP→HP_. In addition, this study proves that causality indexes are clinically relevant given their relation with the quality of life. Remarkably, the association between the strength of the baroreflex involvement and the FIQ score was not detected at REST, thus stressing the importance of challenging cardiovascular control to evoke meaningful responses useful for clinical purposes.

Among the possible limitations of the study there are the limited sample size and the sole inclusion of subjects after complete wash-out. Since increasing the sample size would augment the power of the protocol, future studies should check whether the limited association between traditional cardiovascular control indexes and the FIQ score is primarily the result of the small sample size. Moreover, future protocols should enroll subjects under standard medication to understand whether the use of drugs improves the degree of involvement of cardiac baroreflex and this improvement could finally lead to a better quality of life.

## Conclusions

The study suggests that the lower the degree of cardiac baroreflex involvement during STAND in women with FMS, the higher the impact of FMS on the quality of life. This result stresses the clinical relevance of computing directionality of the interactions along with cardiac baroreflex from spontaneous fluctuations of SAP and HP to provide a quantitative assessment of the state of the FMS patient.

## Supporting information

S1 Dataset (XLSX)
